# Pathological and molecular investigation of infectious bronchitis in broilers: analyzing the impact of biosecurity lapses

**DOI:** 10.3389/fvets.2025.1548248

**Published:** 2025-02-28

**Authors:** Jelena Maletić, Nemanja Jezdimirović, Ljiljana Spalević, Bojan Milovanović, Ana Vasić, Jasna Kureljušić, Branislav Kureljušić

**Affiliations:** ^1^Department of Epizootiology and Health Care of Poultry and Birds, Scientific Veterinary Institute of Serbia, Belgrade, Serbia; ^2^Department of Epizootiology, Clinical Pathology, Pathological Morphology and Reproduction, Scientific Veterinary Institute of Serbia, Belgrade, Serbia; ^3^Department of Bacteriology and Parasitology, Scientific Veterinary Institute of Serbia, Belgrade, Serbia; ^4^Department of Food and Feed Safety, Scientific Veterinary Institute of Serbia, Belgrade, Serbia

**Keywords:** infectious bronchitis, biosecurity, broiler, pathology, farm

## Abstract

**Introduction:**

Infectious Bronchitis (IB) is an acute, highly contagious disease of poultry that leads to significant economic losses in intensive production systems. Preventive biosecurity measures are essential to control its spread, particularly in broiler farms. This study aimed to investigate the relationship between IB outbreaks and biosecurity practices on a broiler farm.

**Methods:**

The farm, housing 96,000 broilers, experienced increased mortality (over 11%) during two consecutive production cycles. Consequently, serological, pathological, molecular and biosecurity investigations were conducted.

**Results:**

Despite a vaccination program using two types of live vaccines (Massachusetts serotype and serotype 793B), serological testing revealed elevated antibody titers against the IB virus, suggesting exposure to a wild viral strain. Necropsy revealed various lesions, including hemorrhagic tracheitis, pulmonary hyperemia, fibrinous pericarditis, splenomegaly, and ascites. Histopathological findings showed necrotic tracheitis, multifocal hepatitis, and purulent bronchopneumonia. By PCR IB viral RNA was detected in all 24 swabs and tissue samples. Biosecurity evaluation revealed significant deficiencies in both external and internal measures, including improper cross-contamination prevention, inadequate flock management, and insufficient vaccination strategies.

**Discussion:**

These biosecurity deficiencies, coupled with the inadequate selection of vaccines not tailored to the prevalent serotypes in the local area, allowed for the introduction and spread of wild IB virus strains. This highlights the critical importance of robust, well-implemented biosecurity protocols in preventing IB on poultry farms.

## Introduction

Infectious bronchitis is a multisystemic disease that primarily affects the respiratory system, but also impacts the urogenital system, leading to kidney dysfunction and decreased egg production, resulting in substantial economic losses in intensive farming (1, 2). According to global economic estimations, infectious bronchitis is one of three diseases that have claimed the largest numbers of losses of animals of different species. Avian infectious bronchitis and LPAI, which caused significant losses, continued to see an increase in losses over time, while none of them exhibited particularly high mobility (3), due to its effects on egg production, shell quality, and hatchability. In broilers, the disease leads to reduced weight gain and increased feed conversion (4, 5).

The causative agent is a virus classified under the family *Coronaviridae*, genus *Gammacoronavirus*, order Nidovirales. Around 30 serotypes of the IB virus (IBV) are known globally, and they differ in their virulence or pathogenicity for respiratory organs, kidneys, or oviducts. Four structural proteins are present in the IBV virion: nucleocapsid, membrane, small membrane, and spike proteins (6). The spike glycoprotein on the virus's surface contains epitopes related to serotype variation and neutralizing antibody binding and is critical for virus attachment and entry into host cells (7). IBV is prone to frequent genetic changes, leading to the continuous emergence of new strains with increased virulence, different tissue tropism, and expanded host range (4). Transmission occurs through respiratory secretions and feces of infected birds. Contaminated equipment and facilities can contribute to spreading the virus from one flock to another if adequate hygiene measures are not implemented (8).

All age categories of poultry are susceptible to infection, and the severity and intensity of the disease are more pronounced in young chicks, with resistance to infection increasing with age (9). The clinical signs depend on which organ systems are affected. Respiratory infection can lead to clinical signs such as gasping, sneezing, lethargy, ruffled feathers, and nasal discharge. Decreased growth and clustering of individuals around heat sources are also observed. In some cases, conjunctivitis, excessive tearing, edema, and cellulitis of the periorbital tissue may occur. The clinical signs in broilers infected with nephropathogenic strains of IBV include depression, watery feces, and excessive water intake. Infection of the reproductive system, particularly damage to the oviduct, results in reduced egg production and quality. Eggs may appear deformed, with rough shells or soft shells containing watery yolks (2). In flocks already infected with immunosuppressive viruses (avian adenovirus, chicken anemia virus, infectious bursal disease virus), the course of IB infection is prolonged, and the clinical signs are more severe. In such cases, the virus can persist in the environment for an extended period, facilitating the development of new genotypes and virus variants (5). Secondary infections with *E. coli* or *Mycoplasma* spp. are common findings in broilers with IB, leading to airsacculitis, increased mortality (10–60%), and higher carcass rejection rates at slaughter (5).

Epidemiological studies have shown that the infection caused by the avian infectious bronchitis virus is endemic in Serbia. Phylogenetic investigations conducted during 2016 and 2017, based on partial S1 protein sequences, revealed the circulation of strains in Serbia classified into the D274 genotype, QX genotype, and 4/91 genotype (10).

Vaccination programs and other biosecurity measures achieve IB control in commercial poultry production. In Serbia, effective live attenuated and inactivated vaccines are available and widely used in practice. However, the virus's tendency to mutate frequently poses a challenge. Many wild IBV and vaccinal strains create an ideal situation for new virus variants (11). To effectively control the situation in a specific area, IB detection, genotyping, and continuous surveillance need to be performed. Factors that predispose IB to increased variability include the introduction of wild genotypes, incorrect vaccine selection and administration, and immunosuppressive diseases (5).

A farm's biosecurity plan includes clearly defined measures aimed at reducing the risk of introducing and spreading pathogenic microorganisms (11, 12) Measures are referred to as external and focused on reducing the risk of pathogen introduction to the farm via humans, equipment, vehicles, wild animals, pets, and other animals, while internal measures aim to reduce the spread of pathogens already present on the farm (12, 13). The occurrence of disease on commercial farms is often associated with failures in the implementation of biosecurity plans, the emergence of new virus serotypes, or improperly executed vaccination programs.

This study aims to investigate the pathological and molecular aspects of an infectious bronchitis case in broiler chickens. Additionally, it seeks to identify failures in biosecurity preventive measures that may have contributed to the outbreak.

## Materials and methods

### Broiler farm

The study was conducted on a broiler farm with a capacity of 96,000 broiler chickens, distributed across four houses. There are no other poultry farms within a 1 km radius of this farm. It is located 2 km away from an artificial lake (fishpond).The farm was built in 2021, and throughout the year, all four houses undergo 5.79 production cycles (batches) successively, with new birds introduced every 63 days. The broilers are normally slaughtered at the age of 38 to 45 days, therefore, each house has a downtime of 10–12 days, but the location where all four houses are situated (the farm) has no rest days until the new chicks are settled in. According to the farm's immunoprophylactic program, broiler chicks were vaccinated on the first day after being delivered on the farm using the spray method with live attenuated vaccines containing two different infectious bronchitis virus serotypes (classic – Massachusetts serotype, strain H-120, GI-1 lineage, and variant—serotype 793B, GI-13 lineage). Depending on the established level of maternal antibodies, the chicks were vaccinated during the second week of life against Newcastle disease virus. The broilers were also vaccinated twice, seven days apart, against the infectious bursal disease virus.

Since the beginning of the 2024 year, during two successive production cycles, high increase of mortality has been observed in two houses of the farm, leading to significant economic losses. The onset of clinical symptoms such as a decrease in food consumption and increased mortality started in the birds aged 2–3 weeks. At the end of the first cycle, mortality in those houses was 11.12%, and at the end of the second, 11.51%. In the other two houses of the farm, the mortality was 7.15% and 7.05%. The total mortality on this farm, or at this location for all four houses, was 9.21%, of which 5.62% of the total number of settled chicks died after 28 days. In this study, ethical approval was not required as it involved routine diagnostic procedures; however, the farm owner consented to publish the results without disclosing the farm's name.

### Clinical examination

During the farm visit, the broiler flock was clinically examined using the standard clinical inspection method (regularly checking the birds for visible signs of illness, injury, or abnormal behavior, that includes assessing their overall health, body condition, posture, and the condition of their feathers, eyes, and respiratory system).

### Serological testing

For diagnostic purposes, serological testing was performed on 40 blood samples from two houses (20 samples per house) collected from the wing veins of 4-week-old chickens using the indirect ELISA method (ID Screen Infectious Bronchitis Indirect Elisa kit, ID Vet, Montpellier, France) during the first and second production cycles. According to the manufacturer's instructions, the antibody titer threshold for a positive-negative result is 1,625, with titers >1,625 considered positive and ≤ 1,625 considered negative.

### Necropsy and histopathological investigation

The carcasses of 12 recently deceased birds, which had previously shown clinical signs of disease, were collected from the second production cycle for necropsy. Following necropsy, trachea, liver, kidney, and proventriculus tissue samples from 4 birds were subjected to histopathological examination, fixed in 10% buffered formalin, routinely processed, and embedded in paraffin blocks. Paraffin sections ~5 μm thick were stained using the hematoxylin-eosin (HE) method.

### Detection and molecular identification of IBV

During the second production cycle, 20 pharyngeal swabs were collected (10 samples per house) from birds exhibiting clinical symptoms for detection and molecular identification of IBV. After necropsy, four tissue samples (trachea, liver, spleen, proventriculus, lungs, kidneys) were also subjected to molecular analysis. Standard bacteriological analysis confirmed presence of *E. coli* in tissue samples (data not shown).

Tissue samples were homogenized using a TissueLyser (Qiagen, Hilden, Germany). Pharyngeal swabs were immersed in 1 ml of sterile PBS and thoroughly vortexed. The tissue and swab suspensions were centrifuged for 1 min at 1,000 x g, and the supernatants were used for RNA extraction (Bioextract Superball extraction kit, Biosellal, Dardilly, France). Real-time RT-PCR for the detection of IBV was performed according to the protocol described by Callison (14) using Luna Universal Probe RT-qPCR Master Mix (NEB, Ipswich, Massachusetts, USA) with a final primer concentration of 0.2 μmol and a probe concentration of 0.1 μmol. Amplification was performed in an AriaMx instrument (Agilent, Santa Clara, California, USA) with the following thermal profile: reverse transcription for 10 min at 55°C, initial denaturation for 1 min at 95°C, and 45 cycles of denaturation at 95°C for 15 s, followed by annealing at 60°C for 1 min.

Part of the hyper-variable S1 gene of IBV was used for Sanger sequencing to determine the genotype of the virus using the Nested RT-PCR protocol described by Worthington (15). The first round of RT-PCR was performed using Luna Universal Probe RT-qPCR Master Mix (NEB, USA) with a final concentration of SX1+ and SX2- primers of 0.2 μmol. Amplification was performed in 2,720 Thermal cycler (Applied Biosystems, USA) using thermal profile: reverse transcription 10 minutes at 55°C, initial denaturation 1 min at 95°C, and 30 cycles of denaturation at 95°C 30 s, annealing at 50°C 1.5 min and elongation at 72°C 2 min, and single step of final extension at 72°C. Second round PCR was performed using OneTaq^®^ Quick-Load^®^ 2X Master Mix (NEB, USA) with a final concentration of SX3+ and SX4- primers of 0.2 μmol. Amplification was performed in 2720 Thermal cycler (Applied Biosystems, USA) using a thermal profile: 30 s at 94°C, and 40 cycles of denaturation at 94°C 30 s, annealing at 48°C 1.5 min and elongation at 68°C 2 min, and single step of final extension at 68°C.

The PCR products were visualized by staining in ethidium-bromide, after electrophoresis in 2% agarose gel. Products with the size of 394 bp were excised from the gel, and purified using a mi-Gel extraction kit (Metabion, Germany), and only one was sent for Sanger sequencing in a commercial company. The consensus sequence was obtained using Chromas lite software.

The molecular testing of samples for the presence of the infectious laryngotracheitis (ILT) virus genome was performed through differential diagnostic procedures. The TaqMan-based real-time PCR was conducted using the commercial kit Quanti Tect Multiplex PCR Kit (Qiagen, Valencia, CA, USA) with the primers and probes that targeted the conserved area of gC gene part of the ILT virus genome, as described by Callison et al. (16).

### Biosecurity assessment

A farm observation and assessment of biosecurity measures and their implementation were carried out. A biosecurity assessment was performed using checklists for broiler farms. The checklist consists of 79 questions divided into 11 categories. External biosecurity was evaluated across eight subcategories: purchase of one-day-old chicks, broiler depopulation, feed and water supply, removal of manure and carcasses, visitors and farm workers, material supply, infrastructure and biological vectors, and farm location. Internal biosecurity was assessed with questions from 3 categories: disease management, cleaning and disinfection, and materials and measures between compartments. Each category was scored from 0 (indicating a complete lack of biosecurity on the farm) to 100 (indicating full implementation of biosecurity measures). The study used the Biocheck.UGent risk-based scoring system (http://www.biocheck.ugent.be) to describe biosecurity assessment on broiler farms. Overall biosecurity was calculated as the average of external and internal biosecurity scores. Veterinarian and farm owner were briefed on the study's goals and procedures before filling the questionnaire. Farm owner provided written informed consent for data collection, sharing, and publication. During the farm visit, we were able to compare the attending veterinarian's and farm owner responses with the actual conditions on the farm and input the correct answers into the questionnaire.

## Results

### Clinical examination

Clinical examination of the flock revealed reduced uniformity, clustering of chickens, and ruffled feathers. Respiratory disease symptoms such as sneezing and nasal discharge were noticed.

### Serological testing

Blood samples revealed a high mean antibody titer against the IBV during two successive production cycles in two farmhouses, indicating that the birds had been exposed to a wild strain of the virus (Table 1).

**Table 1:** Results of IB antibodies dynamics of broiler chicken blood samples in two houses during two successive production cycles (the downtime between two production cycles is < 10 days).

	**Number of samples**	**Mean titer value**	Number of suspect samples^*^	**Highest antibody titer**	**Coefficient of variation (%)**
1st, House 1	20	10,202	18 (90%)	15.951	20
1st, House 2	20	10,338	13 (65%)	16.022	37
2nd, House 1	20	7,494	7 (35%)	15.777	56
2nd, House 2	20	13,372	18 (90%)	15.940	16

^*^Suspect samples are those with antibody titers indicating possible exposure to the wild strain of the IB virus.

**Figure 1:**
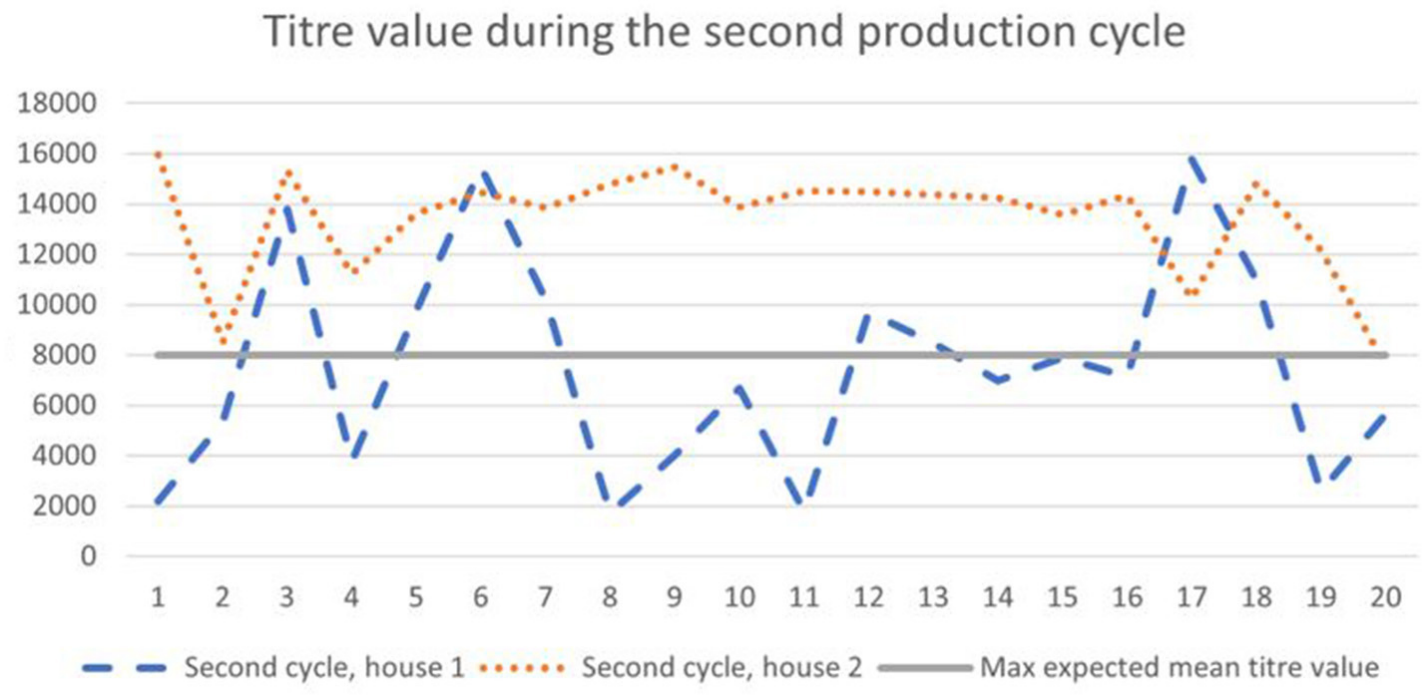
Results of IB antibodies dynamics of broiler chicken during the first cycle with the maximum expected titer value for the applied vaccination program.

**Figure 2:**
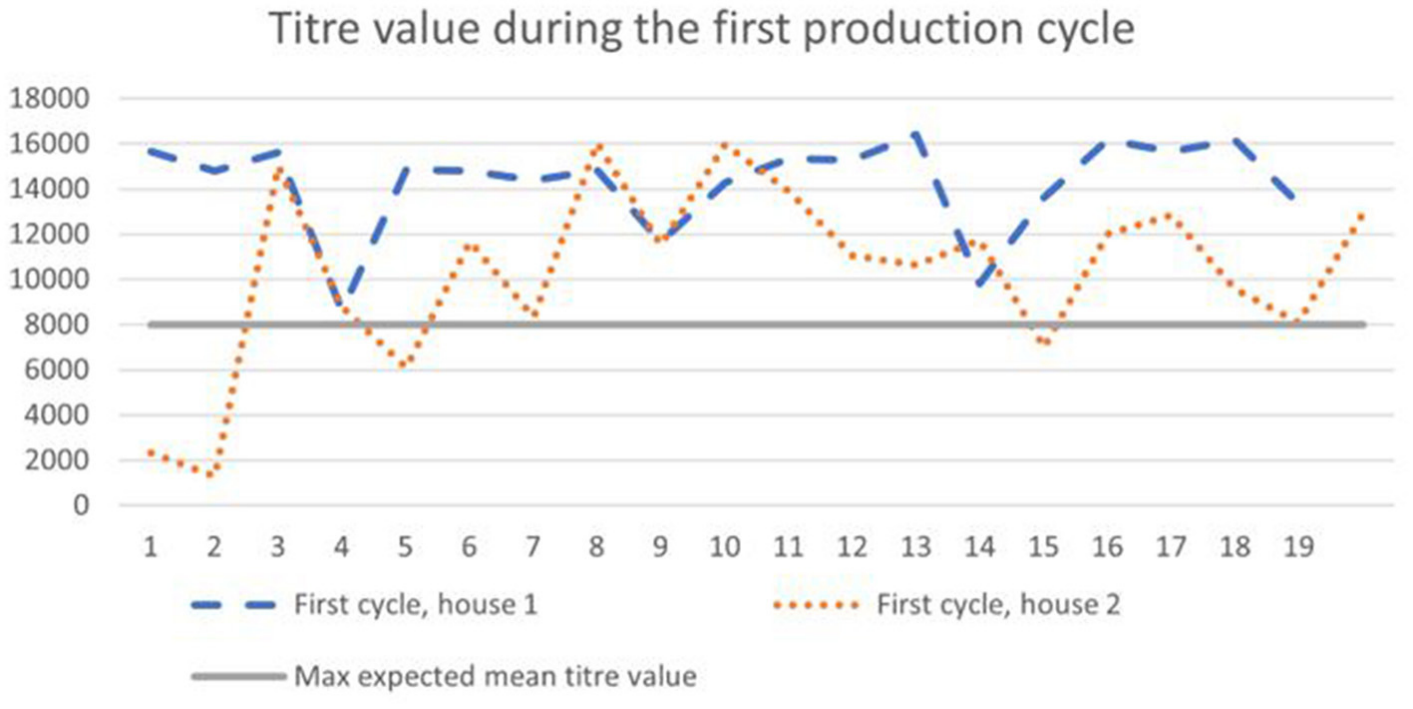
Results of IB antibodies dynamics of broiler chicken during the second cycle with the maximum expected titer value for the applied vaccination program.

According to the general titer baseline of the producer of the ELISA test, the expected mean titer value for a single application of live vaccine (classical plus variant strain), is 4,000–8,000, with a coefficient of variation of 40–80%. During the first cycle, 65–90% of birds (Figure 1) had titer values of more than 8,000, and during the second cycle 35–90% (Figure 2).

### Pathological lesions

**Figure 3:**
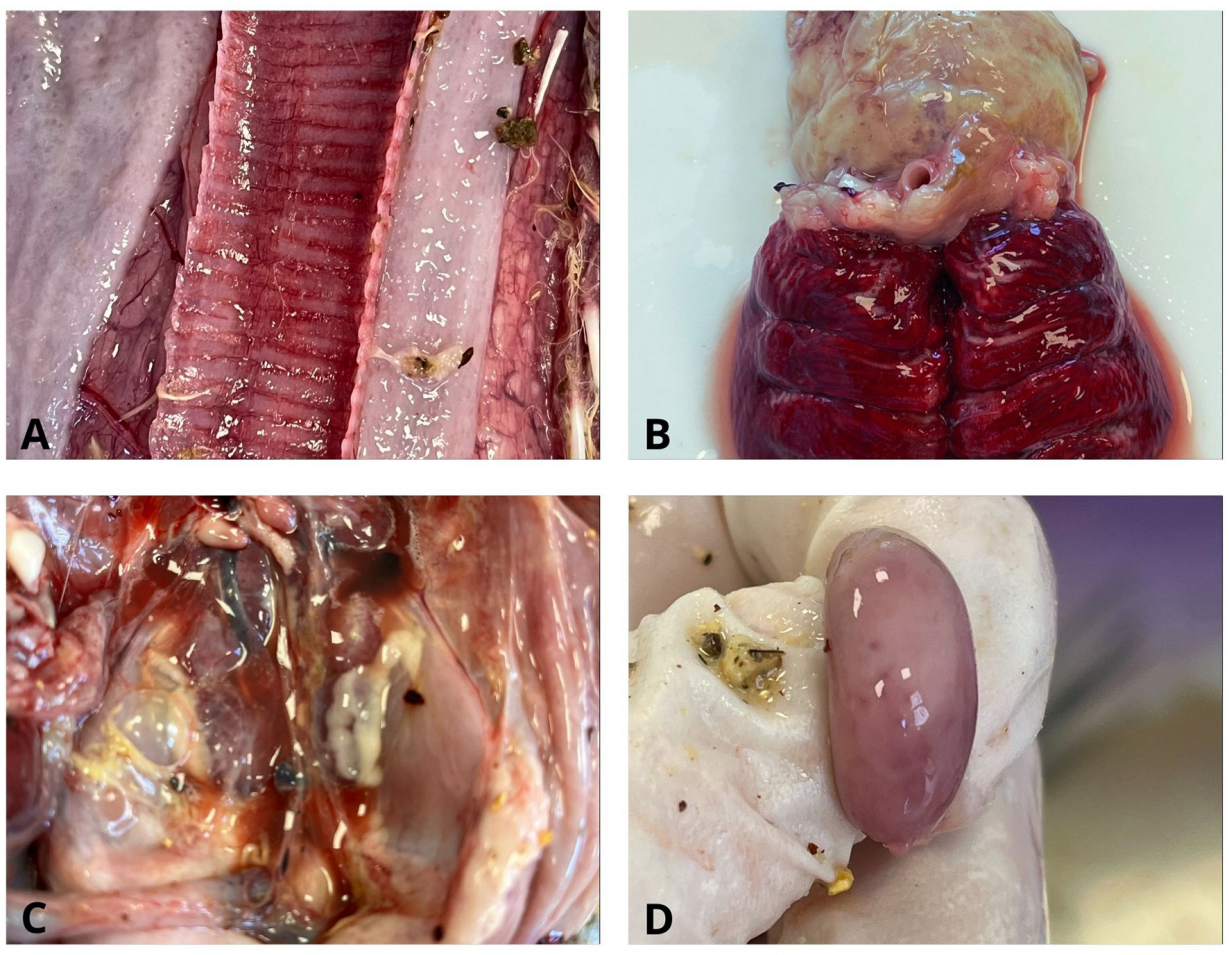
Gross pathological lesions in chickens. **(A)** Trachea. Catarrhal hemorrhagic tracheitis; **(B)** Lung and heart. Pulmonary hyperemia and fibrinous pericarditis; **(C)** Pleuroperitoneal cavity. Nephromegaly, ascites, and fibrinous pleuroperitonitis; **(D)** Splenomegaly with pinpoint splenic hemorrhages.

Gross pathological examination of the broiler carcasses from the second production cycle revealed the following lesions: catarrhal hemorrhagic tracheitis, pulmonary hyperemia, fibrinous pericarditis and perihepatitis, adhesive airsacculitis, splenomegaly with pinpoint splenic hemorrhages, hepatomegaly, nephromegaly, proventriculus dilation, pododermatitis, hemorrhages of the ileocecal tonsils, and ascites (Figure 3).

**Figure 4:**
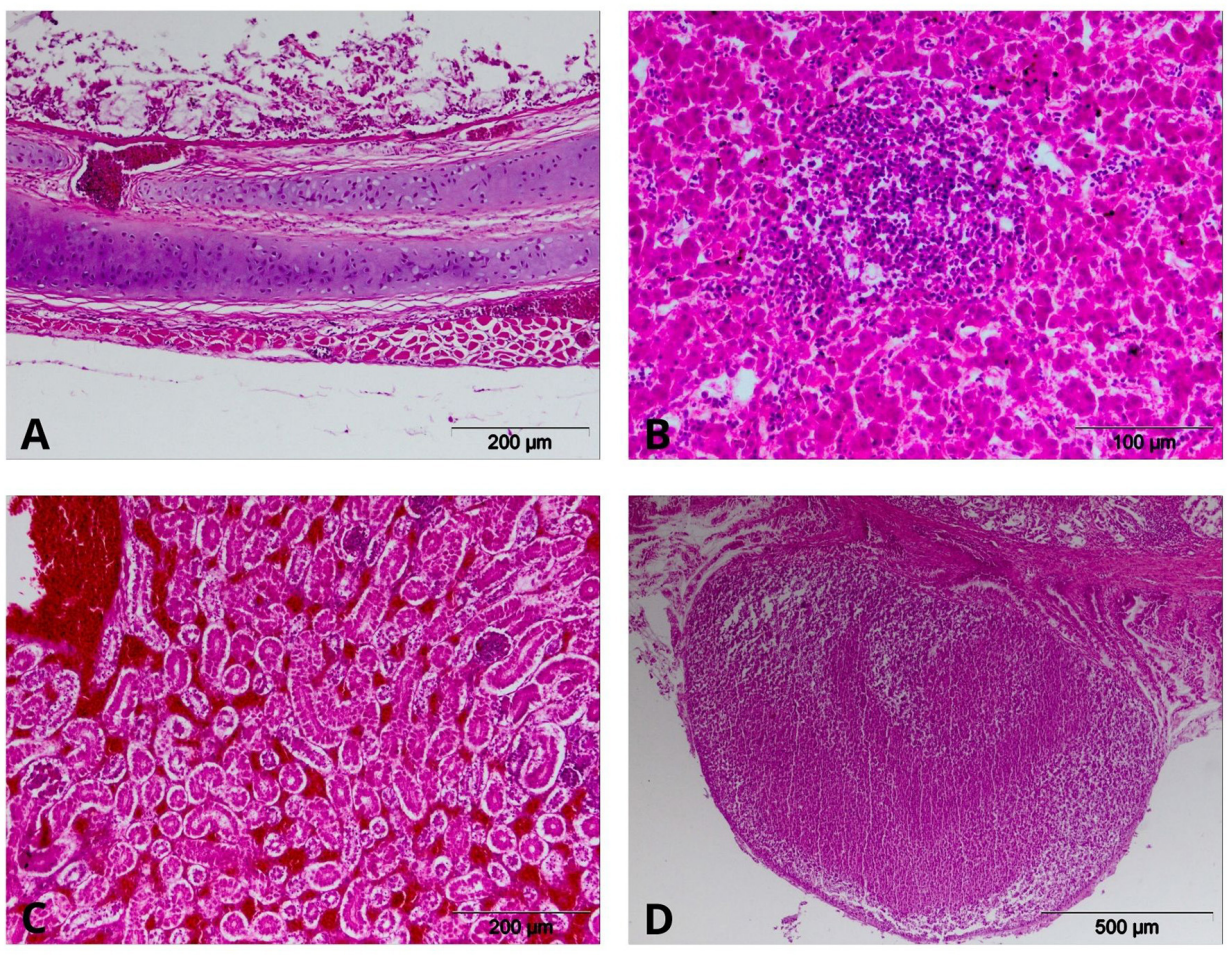
Histopathological lesions in chickens. **(A)** Trachea. Desquamative, necrotic tracheitis; **(B)** Liver. Multifocal lymphoplasmacytic hepatitis; **(C)** Kidney. Hyperaemia, hemorrhages, and tubulonecrosis; **(D)** Proventriculus. Pyogranulomatous proventiculitis (H&E).

Histopathological examinations revealed desquamative necrotic tracheitis, multifocal lymphohistiocytic hepatitis, purulent bronchopneumonia, reactive splenitis, renal hyperemia and hemorrhages with tubular necrosis, and pyogranulomatous proventriculitis (Figure 4).

### Molecular investigation

The genome of the infectious bronchitis virus (IBV) was detected in all 20 pharyngeal swab samples and 4 tissue samples tested, representing 100% of the samples. By comparing the consensus of partial S1 gene nucleotide sequence product with the size of 394 bp with the sequences from the GenBank, it was determined that 100% homology with previously deposited IBV vaccine strain 4/91, GI-13 lineage (NCBI GenBank, Acc. No. KF377577.1). The result of molecular analysis for the detection of the ILT virus genome was negative.

### Biosecurity assessment

Assessing the biosecurity, it was noted that the farm has a biosecurity plan in place, with defined measures related to both external and internal biosecurity. The biosecurity assessment results are presented in Table 2.

**Table 2:** Biosecurity evaluation results on the observed farm.

**Biosecurity assessment**	**Farm (%)**	National average^*^(%)	**Global average (%)**
One-day-old chick purchasing	69	62	67
Broiler depopulation	**43**	48	65
Feed and water supply	67	60	62
Manure and carcass removal	**66**	39	67
Farm workers and visitors	84	73	76
Material supply	**56**	81	70
Infrastructure and biological vectors	97	81	82
Farm location	81	73	68
External biosecurity score	71	65	70
Disease management	**74**	77	80
Cleaning and disinfection	**52**	54	71
Materials and measures between compartments	82	71	75
Internal biosecurity score	66	66	75
Overall biosecurity score	70	65	72

^*^National Average - taken from the Biochek.UGent database, average obtained by completing 46 questionnaires. Bolded values are below the global average.

Critical points in external biosecurity that were identified through observation and assessment include infrastructure, farmworkers and visitors, partial depopulation, and carcass storage.

(a) Crossing of clean and dirty pathways: According to the check list, the farm fulfills all requirements concerning infrastructure. Poultry do not have access to the outside. Wild birds or vermin cannot enter in the house (air inlets are protected). The farm is fenced, the surrounding is clean and paved. Rodent and pets' control is present. However, there was cross-contamination between clean and dirty areas. No distinction between the areas that use external vehicles (e.g., for feed delivery, manure, carcass removal, external transport, etc.) and internal farm movement zones. The farm lacks a place intended for cleaning and disinfection of external vehicles. The farm is fenced, but the gate is always open, without entrance control (no sing that stop the entrance). The external trucks may entrance undisinfected and unwashed.

(b) Farmworkers and visitors: There was no clear notification limiting access to the poultry houses for people (employees and visitors) without prior registration—It is implied, but it is not written or clearly stated anywhere, and it also does not mean that it is always followed. The number of employees with direct access to the poultry was minimized (two persons per shift manage the flocks in two houses—four people for the whole farm). However, there was no zoning of the barn anteroom in relation to changing boots, clothing, and hand sanitation, nor a well-organized sanitary area between different houses where employees could change clothes, shoes, and wash their hands before entering. The anteroom is designed so that workers can follow all the outlined steps for prevention, but the reality indicates a low level of hygiene and adherence to the established rules. Furthermore, we observedbased on the field visit no consistency enforce the protocol for visitors and employees when transitioning from one house to another.

(c) Partial depopulation of broiler: The flocks in each house were partially depopulated in three to four steps. Workers involved in the poultry depopulation process (farmworkers and time-part paid workers from outside) are not provided with specific clothing, shoes, or gloves—there are no measures in place at the beginning of the process. The loading truck arrives empty, cleaned, and disinfected, but the catching is performed by a large number of people who do not apply biosecurity measures for the occasion.

(d) Carcass storage area: Although equipped with cooling and fully enclosed, the carcass storage area is not located in a clearly defined dirty zone of the farm and is not sufficiently distant from the farm's production units. Workers handling carcasses either were not provided with protective gloves or did not use them.

Identified shortcomings in internal biosecurity included the presence of animals of different ages on the same farm, as well as inadequate cleaning and disinfection procedures.

(a) Different ages of poultry: Chickens of different ages are housed in four separate units on the farm. Disease monitoring and vaccinations are conducted regularly. Carcasses of dead birds are removed multiple times a day, but weak and clinically ill birds are not regularly separated, no quarantine. Stocking density ranges from 33 to 39 kg/m^2^.

(b) Cleaning and disinfection protocols: The farm has a specific protocol for cleaning and disinfecting the premises after each production cycle, but the effectiveness of this process is rarely verified. The downtime between two production cycles is < 10 days (from the moment of completed disinfection to the moment of placing a new flock).

## Discussion

Infectious bronchitis (IB) is a significant endemic viral respiratory disease that spreads between farms and between different houses of farm, both in vaccinated and unvaccinated poultry (17, 18). Immunoprophylactic measures are usually carried out according to the vaccination programs in commercial poultry flocks in Serbia (19). Despite the use of live and inactivated vaccines, the occurrence of IB is nearly constant in regions where the infection has already been diagnosed (20).

This study indicated that the farm had repeated IB infection in two subsequent production cycles, which caused huge economic costs for the owner. The IBV's ability to persist in the intestinal tract and feces for several weeks or months, and its shedding through the respiratory system (aerosol) and feces (8, 21), can help to understand the persistence and transmission in the observed farm. On the studied farm, routine monitoring was not performed, and vaccination was provisorily done. Regular monitoring of the local situation establishes baseline antibody levels, which are then used for comparison. It is also essential to monitor and record data from specific environments (e.g., farms, and regions) to understand the normal range of antibody levels, as antibody levels in the blood can vary due to different rearing conditions (21). Enzyme-linked immunosorbent assays (ELISAs) represent an appropriate methodology test for routine IB monitoring due to their cost-effectiveness and quick turnaround of results (22). ELISA tests also are the preferred diagnostic approach for the detection of antibodies in poultry flocks resulting from either infection or vaccination (23, 24). High, uniform, and long-lasting antibody titers indicate a well-conducted and adequate vaccination. Low, uneven, and short-lasting titers suggest that vaccination was unsuccessful, likely due to improper application or poor vaccine quality. According to the obtained results, the mean level of antibodies was almost twice as much as expected for the applied vaccination program and the coefficient of variation was below 20%, which means it is uniform in the flock and that we can expect similar results for all birds. If titers are significantly higher than expected, a field infection should be suspected, meaning that the detected antibody levels in the blood are not the result of a regular vaccination program but rather exposure to a wild field strain of the virus (25, 26). According to studies by Leerdam (27) and Bhuiyan (28), average titer values after infection should increase significantly, by at least twofold, compared to post-vaccination levels or pre-infection baseline titers. It is common for antibody titers to rise sharply 3–4 weeks after the onset of infection (28). According to previous investigations, smallholder farms in Serbia are facing a high prevalence of IBV. The worrying fact is that only 5% of non-vaccinated flocks were negative for IBV (19). In the present study, the level of antibody titers, the number of suspect samples, and the coefficient of variation (Table 1) may indicate that the birds had been in contact with a wild virus strain.

In this study, the wild-type virus could have been present, causing increased clinical signs. The IBV genome was detected using molecular methods, and *in silico* compared with wild strain. We were not able to prove presence of wild type virus due to sample size, which is a limitation of this study. In this case, a wild strain was likely present in the samples, although it was not identified through sequencing. Had we sequenced a larger number of different samples, maybe it would have been detected. Also, another limitation in this case was the limited length of 394 bp of the S1 gene that was observed which cannot reflect the whole molecular characteristics of the virus (29). Furthermore, the observed gross lesions and histopathological findings, combined with serological analysis of blood samples and detection of the virus genome in the swab samples and internal organs, consistent with findings from other researchers (26), suggest a strong confirmation that the birds were infected with IBV during the second production cycle. Detected pathological lesions are very suggestive for IB, however impact of other infectious agents such as *Mycoplasma* cannot be excluded. Given that serological analysis of blood samples from both houses during the first cycle also showed significantly high average antibody titers, it can be suspected that the birds had been exposed to a wild strain during the previous cycle, exposing the facility to the IBV.

Controlling IB and other infectious diseases in broilers can be enhanced through good farm management, appropriate stocking densities, quality air, and extended downtime between production cycles (30). The cornerstone of preventive measures in the fight against IB is biosecurity. This involves the strict implementation of external and internal biosecurity measures to regulate the movement of animals, people, materials, and waste (31). Assessing biosecurity protocols on broilers' farms is a useful tool for identifying potential risks, preventing the introduction or spread of diseases, and improving overall flock health and productivity. Some pathogens can serve as a biomarker for the efficiency of the implemented biosecurity protocols (32). According to the regulation, in Serbia, owners and animal keepers ensure animal health and wellbeing, taking measures to prevent the spread of infectious diseases, including biosecurity and good farming practices. Critical biosecurity points include: the farm location, physical visibility of the farm and its separation from the surroundings, movement of people, movement of animals and vehicles within and outside the farm, food and medications brought in and used, animal reproduction using artificial insemination or natural mating, handling of animal carcasses, handling of waste water and manure from the farm, pest control, and the conditions of the facility, equipment, and microclimate (33) In this study, the assessment of biosecurity measures on the farm identified critical points in external biosecurity measures (cross-contamination of clean and dirty pathways, thinning management, protocols for staff and visitors, procedures for manure and carcass disposal), as well as internal biosecurity measures (immunoprophylactic programs, stocking density, the presence of different age groups of chickens at the same location). Given the IBV transmission pathways and their ability to survive for extended periods, along with the identified deficiencies in biosecurity measures, it can be assumed that these factors significantly contributed to virus transmission between houses and production cycles. In four different houses (1 to 4), the farm has the presence of different age groups of chickens. In one moment, they may have the depopulation of house number 4 and one-day chicken introduction in house number 1. This increases the risk of the virus spreading. Also, stocking density on farms affects the risk level of virus transmission between farms. However, it has not been determined whether this facilitates virus transmission via air due to proximity or due to shared risk factors (horizontal contacts or environmental conditions) (25, 28). The severity of early-age IB infections can be controlled by reducing extreme amounts of ammonia, carbon dioxide, and hydrogen sulfide, and by maintaining environmental temperature according to the technology prescribed for that age and provenience. The ammonia concentration in the poultry houses above 25 ppm can cause damage in productive performance, impair immune response (34) and disturb the respiratory system of broilers (35, 36) so the broiler may become more susceptible to viral infections (28, 37). Also, it is reported that high level of ammonia can lead to low breast muscle and carcass composition in broilers (38) Studies showed that the downtime period between production cycles should be not < 14 days and the proper performing of procedures of cleaning, washing, and disinfection are essential to reduce the risk of infection in the next production cycle (39, 40).

Concerning IB infection, vaccination as one of the measures of internal biosecurity, is considered the most effective and widely used preventive measure. Although vaccination cannot completely prevent infection, it can reduce clinical symptoms and infection pressure (41). Due to the short life of broilers, they are vaccinated once or twice against IB. In practice, broilers are usually vaccinated with spray vaccines containing 1–3 serotypes of live attenuated vaccines immediately after hatching, or still in the incubator before transport to the farm. For broilers whose production cycle lasts longer than 49 days and in facilities with an increased risk of infection, an additional dose of live attenuated vaccine is administered through drinking water between the 14th and 18th days of age to extend immunity (5, 42, 43).

Eradicating IB remains a challenging goal. The results obtained showed the internal biosecurity score was lower than the external biosecurity score. This is not a common finding for broiler farms (31, 44, 45). Enhancing internal biosecurity frequently requires the implementation of fundamental interventions within the flock, including the establishment of stringent hygiene protocols and adherence to appropriate operational procedures (46). Previous studies showed that broiler farms with better overall biosecurity programs and management practices have a lower risk of transmission of immunosuppressive pathogens (47). Field efforts should be directed toward optimizing the application of well-established, effective measures. Additionally, continuous monitoring, based on objective criteria and knowledge of the local epidemiological status, is crucial (25).

## Conclusions

In this study, infectious bronchitis (IB) was confirmed through clinical, serological, molecular, and pathological examinations, highlighting notable deficiencies in biosecurity measures. The failure to properly implement the biosecurity program, combined with the use of vaccines not based on continuous monitoring of the most prevalent serotypes in the local area, may have contributed to the outbreak, potentially due to the introduction of wild IB virus strains, as suggested by the findings. However, it is important to note that the study is based on a single farm, and the authors cannot be entirely certain about the role of wild strain infection. Since no vaccine can offer complete protection without effective biosecurity practices, it is crucial to provide farmers with proper guidance on the implementation of preventive measures. Additionally, regular monitoring of major viral diseases should be emphasized. Based on these observations, a tailored vaccination strategy for each farm is recommended to improve disease control.

## Data Availability

The original contributions presented in the study are included in the article/supplementary material, further inquiries can be directed to the corresponding author/s.
